# Pectic Polysaccharides Recovery from Rapeseed Meal *via* Conventional and Enzyme-Assisted Extraction Techniques: Toward Emerging Prebiotic Pectic Oligosaccharide Development

**DOI:** 10.3390/foods15081338

**Published:** 2026-04-12

**Authors:** Katarina Banjanac, Milica Veljković, Milica Simović, Aleksandra Tomić, Paula López-Revenga, Antonia Montilla, Francisco Javier Moreno, Dejan Bezbradica

**Affiliations:** 1 Innovation Center of Faculty of Technology and Metallurgy, Karnegijeva 4, 11120 Belgrade, Serbia; kbanjanac@tmf.bg.ac.rs; 2 Faculty of Technology and Metallurgy, University of Belgrade, Karnegijeva 4, 11120 Belgrade, Serbia; mcarevic@tmf.bg.ac.rs (M.S.); atomic@tmf.bg.ac.rs (A.T.); dbez@tmf.bg.ac.rs (D.B.); 3 Grupo de Química y Funcionalidad de Carbohidratos y Derivados, Instituto de Investigación en Ciencias de la Alimentación, CIAL (CSIC-UAM), 28049 Madrid, Spain; p.lopez.revenga@csic.es (P.L.-R.); a.montilla@csic.es (A.M.); javier.moreno@csic.es (F.J.M.)

**Keywords:** rapeseed meal, enzyme-assisted extraction, pectic polysaccharides, pectic oligosaccharides, prebiotics

## Abstract

This study investigates the extraction of pectic polysaccharides from rapeseed meal (RSM) using both conventional and enzyme-assisted techniques, and the obtained pectic polysaccharide fractions will be used later to produce prebiotic pectic oligosaccharides (POS). A two-step process was developed, involving enzymatic treatment with Alcalase^®^ 2.4 L for 2 h and Cellic^®^ CTec3 HS preparations for 24 h, followed by ammonium oxalate extraction, which effectively isolated two pectic polysaccharide-enriched fractions: PP-EAE (first step) and the resulting Ca-bound pectic polysaccharides fraction (CaPP-EAE) (second step). Both fractions exhibited a bimodal molecular weight profile, indicative of the presence of long-chain polysaccharides alongside oligosaccharides. CaPP-EAE compositional analysis revealed that the fraction contained 56.8% galacturonic acid (GalA), low methyl-esterified (LM) pectins with 53.2% homogalacturonan (HG) and 30.2% rhamnogalacturonan I (RG-I) domains, featuring side chains of arabinan, arabinogalactan, and galactan. Subsequent enzymatic treatment with 0.5% (*v*/*v*) of Pectinex^®^ Ultra Passover for 30 min transformed these fragments into a mixture of short-chain POS. Importantly, the produced short-chain POS fraction demonstrated enhanced prebiotic activity, particularly for bacterial strains of the family *Lactobacillaceae*, compared to a yeast strain. These findings provide a sustainable, biorefinery-compatible approach for extracting and modifying RSM polysaccharides, supporting the development of structurally defined POS as novel prebiotics.

## 1. Introduction

The extraction of pectin from agro-industrial biomass presents a sustainable and economically viable solution to address both environmental pollution and the rising demand for natural polymers. Historically, pectin production was driven by the abundance of fruit-processing residues from the juice industry, especially citrus peel [1,2] and apple pomace [3], which have emerged as valuable sources of this versatile biopolymer widely used in the food, cosmetic, and pharmaceutical sectors [1]. Pectin is a complex family of polysaccharides composed mainly of homogalacturonan (HG), rhamnogalacturonan I (RG-I), and rhamnogalacturonan II (RG-II). RG-I consists of a repeating disaccharide backbone of rhamnose (Rha) and galacturonic acid (GalA) [→2)-α-L-Rha-(1→4)-α-D-GalA-(1→], with many rhamnose residues linked to neutral sugar side chains such as arabinan, galactan, and arabinogalactans. HG is a linear polymer of α-(1→4)-linked D-GalA, while RG-II is considered the most structurally complex region of pectin, consisting of a short HG backbone with several highly branched side chains that include rare sugars [4]. The relative proportions and fine structure of these domains strongly influence pectin’s gelling, thickening, and stabilizing properties, as well as its recognition as a dietary fiber with documented health benefits [5]. Beyond its traditional role, the growing evidence indicates that pectin represents a source of bioactive derivatives such as pectic oligosaccharides (POSs), which have recently attracted interest in medical research for their potential as novel prebiotic compounds [6,7]. POS usually consist of 2–50 monomeric units or have molecular weights reaching 5–7 kDa and are obtained by pectin depolymerisation [8]. Several studies reported that POSs modulate gut microbiota composition, promote satiety, exhibit selective cytotoxicity with potential anti-colon cancer effects, support cardiovascular health, and reduce levels of insulin and gastric inhibitory polypeptide (GIP) [6,9,10]. However, the biological activity of POSs is strongly dependent on the structural features of the parent pectin, which in turn are determined by origin and extraction method, as well as hydrolysis method. This dependence, together with increasing consumption of pectin, underscores the need to explore new biomass sources and new, more efficient extraction strategies capable not only of enhancing pectin yield but also of obtaining structurally diverse pectins suitable for targeted biotransformations to POSs [4,5].

Rapeseed meal (RSM), a major by-product of canola oil production, represents an underexploited agro-industrial by-product. It holds significant industrial potential owing to its large reserves (over 25 million tons are produced annually) and its rich nutritional profile, which includes more than 30% proteins, 30–60% fibers, carbohydrates, and different bioactive compounds [11]. To date, it has been primarily used for agricultural purposes, including organic fertilizer and animal feed. However, its wider application is limited by antinutritional factors, such as glucosinolates, phytic acid, and phenolic derivatives that impair nutrient absorption, especially when used as animal feed [12]. From a biorefinery perspective, RSM holds particular promise because its carbohydrate fraction contains a relatively high proportion of pectin compared to other polysaccharides and other oil crops as well [12]. Yet, most valorization studies have focused on cellulose and hemicellulose, leaving the dominant pectin fraction largely uncharacterized [12] and its potential as a precursor for POSs is unexplored.

Since RSM contains a higher level of pectin compared to other polysaccharides [12,13], investigating extraction methods for pectin could improve processing efficiency and boost economic value. Traditional extraction methods involve the use of strong mineral acids such as nitric, sulfuric, phosphoric, or hydrochloric acid combined with heat [14]. While mineral acids are effective for pectin extraction, their corrosive nature raises environmental and economic concerns. As a result, the industry is shifting towards greener methods, using milder organic acids like acetic and citric acid. Although less aggressive, these organic acids provide a more sustainable and environmentally friendly alternative. Traditional acid-based extraction methods face challenges such as extreme extraction conditions (pH and temperature), and pectin degradation. To overcome these, green extraction techniques have been developed to enhance efficiency, preserve pectin’s functional properties, and minimize the solvent use. These include methods like microwave-assisted [3], enzyme-assisted, ultrasound-assisted, subcritical fluid, high hydrostatic pressure, deep eutectic solvent extraction, and various combinations of these approaches [14,15].

This study aims to identify the most effective methods for extracting pectic polysaccharides from rapeseed meal (RSM). Three extraction approaches were compared: conventional mineral acid extraction (HCl), organic acid extraction (citric acid), and enzyme-assisted extraction (EAE) using two commercial enzyme preparations (Alcalase^®^ 2.4 L and Cellic^®^ CTec3 HS, Novozymes, Bagsvard, Denmark). The citric acid and EAE methods were further combined with a secondary extraction step using ammonium oxalate as a chelating agent. The efficiency of these approaches was evaluated based on extraction yield and the physicochemical properties of the resulting polysaccharide fractions. The central hypothesis was that targeted EAE, particularly using enzyme systems with defined cellulolytic, hemicellulolytic, and proteolytic activities, can selectively disrupt the RSM cell wall and enhance the release of structurally distinct pectic polysaccharides. This approach is expected to improve extraction efficiency compared to conventional acid-based methods and allow modulation of pectin molecular characteristics through controlled enzymatic action. Furthermore, it is hypothesized that enzymatic hydrolysis of a pectin-enriched fraction using Pectinex^®^ Ultra SP-L, Novonesis, Lyngby, Denmark will produce low-molecular-weight pectic oligosaccharides (POS) with prebiotic potential, providing added functional value to RSM-derived ingredients.

## 2. Materials and Methods

### 2.1. Materials

The rapeseed meal (RSM) utilized in this study was a kind donation from Victoriaoil Ltd. (Šid, Serbia). Analytical-grade chemicals, including ethanol, acetone, hydrochloric acid, citric acid, and ammonium oxalate, were obtained from Centrohem (Stara Pazova, Serbia). HPLC-grade solvents (acetonitrile and water), tri-fluoroacetic acid (TFA), hydroxylamine chloride, pyridine, hexamethyldisilazane (HMDS), pullulan standard set, phenyl-*β*-D-glucoside, and 1-phenyl-3-methyl-5-pyrazolone (PMP) were purchased from Sigma-Aldrich (Schnelldorf, Germany). The enzymes used in the study were Alcalase^®^ 2.4 L (Alcalase^®^) and Cellic^®^ CTec3 HS (Cellic^®^), Novozymes (Bagsvard, Denmark), and Pectinex^®^ Ultra Passover (Pectinex^®^), Novonesis (Lyngby, Denmark).

### 2.2. Pectic Polysaccharides Fraction Isolation

**Figure 1:**
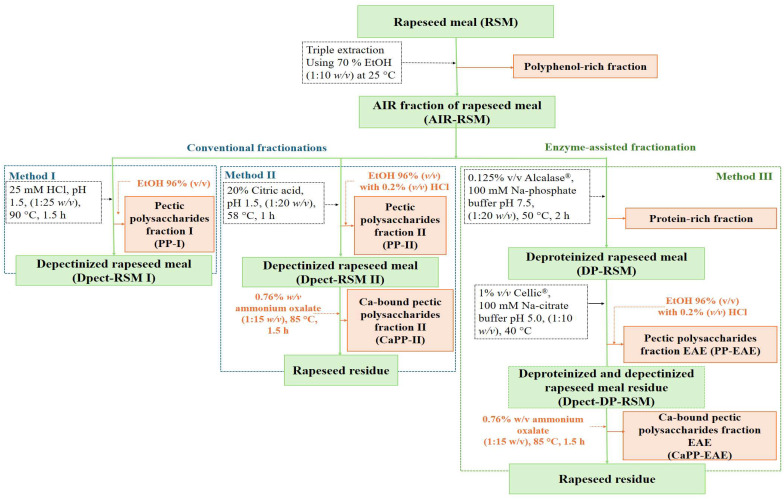
Overview of applied treatments for isolation of pectic polysaccharides-enriched fractions from RSM.

The schematic overview of the pectic polysaccharides extraction procedures applied to RSM is depicted in Figure 1. Initially, the alcohol-insoluble residue from RSM (AIR-RSM) was prepared by performing three successive extractions with 70% (*v*/*v*) aqueous ethanol at a solid-to-solvent ratio of 1:10 (*w*/*v*). The solid residue was then recovered *via* vacuum filtration and washed sequentially with 96% ethanol and acetone.

**Conventional HCl acid extraction (Method I, Figure 1)**. The applied extraction procedure was described in our previously published study [16]. Concretely, the AIR-RSM was mixed with approximately 25 mM HCl (pH adjusted to 1.5) at a 1:25 (*w*/*v*) ratio, heated to 90 °C for 1.5 h. The resulting extract was separated by vacuum filtration, and the solid residue was rinsed with hot water. Pectic polysaccharides-enriched fraction I (PP-I) was then precipitated from this mixture by overnight ethanol precipitation at 4 °C, using 4 volumes of 96% ethanol, and recovered by centrifugation at 4430 *g* for 10 min.

**Conventional citric acid extraction (Method II, Figure 1)**. The applied extraction procedure using citric acid followed the protocol described in the study of Ćorović et al. [17] The extraction mixture was prepared by mixing AIR-RSM with a 20% solution of citric acid to reach a pH value of 1.5 at 1:20 (*w*/*v*). This mixture was kept in a water bath at 58 °C for 1 h. Pectic polysaccharides fraction (PP-II) was then precipitated from this mixture by ethanol precipitation overnight at 4 °C, using 4 volumes of 96% ethanol with 0.2% (*v*/*v*) HCl, and recovered by centrifugation at 4430 *g* for 10 min. The solid residue (Dpect-RSM II) was washed with 96% ethanol and acetone, then dried. The Dpect-RSM II was used as a starting material for the extraction of Ca-bound pectic polysaccharides (CaPP-II).

**Enzyme-assisted extraction with Alcalase^®^ 2.4 L and Cellic^®^ CTec3 HS (Method III, Figure 1)**. In the first stage of the enzyme-assisted extraction (EAE) process, using the commercial protease preparation Alcalase^®^, AIR-RSM was suspended in 100 mM sodium phosphate buffer (pH 7.5) at a ratio of 1:20 (*w*/*v*). The enzymatic reaction was initiated by adding 0.5% (*v/w*) Alcalase^®^, followed by incubation with constant stirring at 50 °C for 2 h. The resulting solid residue, deproteinized RSM (DP-RSM), was collected by vacuum filtration, rinsed immediately with distilled water, and further washed with 96% ethanol and acetone to remove residual enzyme and halt the reaction, respectively. The detailed procedure is described in our previously published study [16].

For the second stage of the EAE process involving the use of efficient cellulase and hemicellulase complex Cellic^®^, DP-RSM was mixed with 100 mM Na-citrate buffer at pH 5.0 at a ratio of 1:20 (*w*/*v*), and then 1% (*v*/*v*) Cellic^®^ preparation was added. The EAE was conducted at 40 °C for 8 and 24 h with constant shaking (200 rpm). After extraction, the samples were heated at 100 °C for 5 min to inactivate the enzyme and then cooled to room temperature. The resulting pectic polysaccharides-enriched fraction (PP-EAE) was separated by vacuum filtration and then treated as PP-II.

**Extraction with ammonium oxalate.** To extract the calcium-bound pectic polysaccharides (CaPP), the solid residues obtained through the extraction Method II and EAE were treated with the chelating agent ammonium oxalate. The solid residues (Dpect-RSM II and one obtained after treatment with Alcalase^®^ (deproteinization) and Cellic^®^ (depectinization) (Dpect-DP-RSM)) were mixed with a 0.76% (*w*/*v*) ammonium oxalate solution in a 1:15 (*w*/*v*) ratio. The mixture was then incubated at 85 °C with agitation for 90 min, following the protocol previously outlined by Ma et al. and Ćorović et al. [17,18]

### 2.3. Enzymatic Production of Pectic Oligosaccharides (POSs)

The obtained CaPP-EAE fraction was ultimately used to obtain POSs. The reaction mixtures were prepared by dissolving the fraction in 100 mM sodium acetate buffer (pH 4.5) to achieve a concentration of 4% (*w*/*v*). The enzymatic reaction was carried out using Pectinex^®^ at a concentration of 0.5–1% (*v*/*v*) at 50 °C, with continuous orbital shaking at 120 rpm. The reactions were monitored for 2 h. At designated time points, aliquots were withdrawn and heated at 100 °C for 5 min to halt the reaction. Portions of these samples were analyzed *via* High-Performance Size-Exclusion Chromatography coupled with an Evaporative Light-Scattering Detector (HPSEC-ELSD) [16].

### 2.4. Pectic Poly- and Oligosaccharides-Enriched Fraction Analyses

**Determination of Monomeric Composition.** Monomeric composition of AIR-RSM and the pectic polysaccharides-enriched fractions was determined using a previously established GC-FID method [19]. A detailed procedure is described in our previously published paper and the Supplementary Information file [16]. Based on the monomeric composition, various structural parameters were calculated (Supplementary Information file), following the methods previously described [17].

**Determination of the Molecular Weight Distribution.** Molecular weight (M_w_) distribution was determined following a previously reported method by Muñoz-Almagro et al. [20].

**Fourier-Transform Infrared Spectroscopy (FTIR) Analysis.** An FTIR analysis of extracted pectic polysaccharides-enriched fractions was performed. A detailed procedure was described in the Supplementary Information file.

**Determination of protein content.** Protein content in the samples was determined by the Kjeldahl method [21].

**Prebiotic activity assay.** The effect of varying concentrations (0.0625–1.0% (*w*/*v*)) of the obtained POSs fraction on the growth of probiotic strains and an indicator strain of a potentially pathogenic bacteria were evaluated using microtiter plates. Different dilutions of the POSs fraction were prepared with appropriate media: nutrient broth for *Escherichia coli* ATCC 25922, MRS without carbohydrates for *Lactiplantibacillus plantarum* 299v and *Lacticaseibacillus rhamnosus* GG, and YPD without carbohydrates for *Saccharomyces boulardii* CBS 5926. Colonies from freshly prepared agar plates were incubated overnight in the corresponding culture media under defined conditions. The experiments were started by adding a defined amount of inoculum in the adequate media with POSs fraction to achieve an initial cell concentration of 1 × 10^7^ for lactic bacteria and *E. coli* and 1 × 10^5^ CFU/mL for *S. boulardii*. The effect of the obtained fractions on the tested bacteria and yeast was evaluated after 24 h of incubation (37 °C for bacteria and 30 °C for yeast). The optical density was measured at a wavelength of 600 nm at 0 and 24 h using a microtiter plate reader (BioTek, Santa Clara, CA, USA). The obtained value were converted in CFU/mL, using the strain—specific calibration slopes previously determined and reported in our earlier study (*E. coli*: 5 × 10^−10^ mL/CFU, *L. plantarum*: 3 × 10^−9^ mL/CFU, *L. rhamnosus*: 5 × 10^−9^ mL/CFU, and *S. boulardii*: 2 × 10^−7^ mL/CFU) [22]. The prebiotic activity was assessed through the prebiotic capacity (PC), representing the growth ratio of probiotic cultures in the presence of POSs fraction relative to pathogenic culture, was calculated following the equation [23]:

$$\mathrm{Prebiotic}\;\mathrm{capacity}\;(PC)=\frac{PB\;24\;h-PB\;0\;h}{PBC\;24\;h-PBC\;0\;h}-\frac{EC\;24\;h-EC\;0\;h}{ECC\;24\;h-ECC\;0\;h}$$

*PB*—Probiotic member with tested sample (prebiotic) and without additional carbon source, CFU/mL
*PBC*—Probiotic member without a tested sample (prebiotic) and carbon source, CFU/mL
*EC*—*E. coli* with tested sample (prebiotic) and without additional carbon source, CFU/mL
*ECC*—*E. coli* without a tested sample (prebiotic) and carbon source, CFU/mL

Positive PC values indicate that the tested compound positively affects the growth ratio of probiotic microorganisms (*L. plantarum*, *L. rhamnosus*, and *S. boulardii*), emphasizing its prebiotic potential. Conversely, negative PC values suggest increased growth of pathogenic bacteria (*E. coli*), while values close to zero imply that the extract has no impact on probiotic bacterial growth.

### 2.5. Statistical Analysis

**Figure 2:**
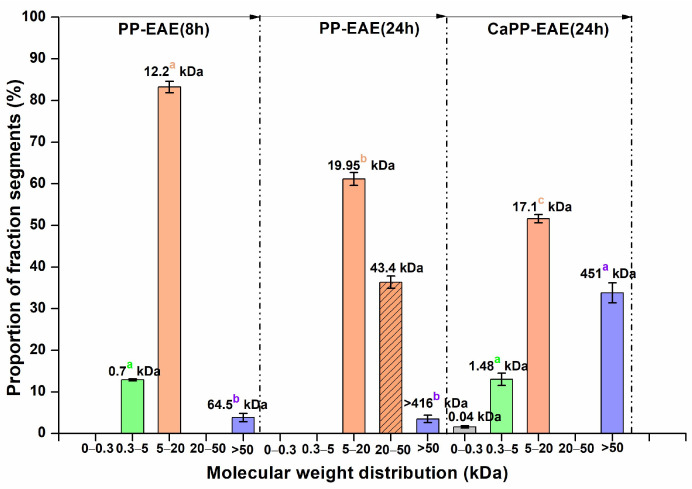
Distribution of Molecular weight (M_w_) by HPSEC-ELSD of the obtained fraction using enzyme-assisted extraction with Cellic^®^ for 8 h and 24 h, and additional extraction using ammonium oxalate: PP-EAE(8 h); PP-EAE(24 h); and CaPP-EAE(24 h). Values on the graph represent the average M_w_ of the fraction fragment. Different characters (e.g., a, b, and c) on the graph at the same average M_w_ distribution range indicate a statistically significant difference (*p* < 0.05) between the proportions of the fraction fragments for each obtained EAE fraction (PP(8 h), PP(24 h), and CaPP(24 h)).

All experiments were conducted in triplicate, except results presented in Figure 2, which were performed in duplicate, and results are presented as mean ± standard deviation. Statistical significance was assessed using one-way ANOVA followed by Tukey’s post hoc test (OriginPro 8.5), with *p* < 0.05 considered statistically significant. Before applying one-way ANOVA, the assumptions of normality and homogeneity of variances were evaluated using the Shapiro-Wilk and Levene’s tests, respectively. Specifically, one-way ANOVA was conducted on the following variables: extraction yield of each polysaccharide fraction, monosaccharide composition, molecular weight distribution parameters, and prebiotic capacity.

## 3. Results and Discussion

Given that polysaccharide composition may differ among species within the Brassica (*Brassicaceae*) family and can be influenced by processing conditions applied during oil production, preliminary compositional analyses were conducted to characterize the rapeseed meal (RSM) obtained after removal of extractable components (including polyphenolic compounds, low-molecular-weight sugars, and pigments). In this study, the resulting alcohol-insoluble residue (AIR-RSM) was used as the starting material for subsequent polysaccharide extraction. The AIR-RSM contained 91.1% dry matter, with proteins accounting for 42.9% of the dry mass (Supplementary Information file, Table S1). Analysis of monomeric composition (Table 1) revealed a moderate galacturonic acid (GalA) content (9.7%), indicative of the presence of pectic polysaccharides. Among the neutral sugars, arabinose (Ara, 42.8%), galactose (Gal, 17.1%), and xylose (Xyl, 15.7%) were most abundant, reflecting contributions from hemicelluloses and probably pectic rhamnogalacturonan I (RG-I) side chains, bearing in mind the rhamnose (Rha, 4.8%) presence. Overall, the compositional profile aligns closely with the values previously reported for *Brassica campestris* seed cake defatted with hexane and acetone [24] and rapeseed (*B. napus*) meal [25]. The main difference is the glucose (Glc) content, but it should be noted that our results are underestimated due to the low efficiency of TFA in hydrolysing cellulose. The obtained results confirm AIR-RSM as a suitable substrate for pectic polysaccharides extraction.

### 3.1. Pectic Polysaccharides-Enriched Fraction Extraction

Three different methods were employed for the isolation of pectic polysaccharides from RSM, as outlined in Figure 1. Since the extraction solvent and parameters such as temperature, pH, and duration strongly influence both yield and quality of extracted fractions, the primary objective was to identify the most suitable extraction approach for this agro-industrial by-product. As mentioned earlier, before application of different extraction methods, AIR-RSM (Figure 1) was obtained by three step extraction using aqueous ethanolic solution (70% (*v*/*v*)), as described in our previously published protocol [16].

#### 3.1.1. Conventional Acid Extraction

Methods I and II involved conventional solid–liquid extraction using HCl or citric acid, respectively. In both cases, polysaccharides were solubilized and subsequently recovered by ethanol precipitation. In addition, the solid phase remaining after citric acid treatment was further treated with a chelating agent (ammonium oxalate) to extract Ca-bound pectic polysaccharides. These methods have been widely reported for pectin extraction from fruits and pomace [17,26]. The applied experimental conditions for these extraction methods were based on the procedures described in the studies by Simović et al. [16], and Ćorović et al. [17], respectively.

The pectic polysaccharides fraction extraction yields on a mass basis obtained using the different conventional acid methods (Method I and II) are summarized in Table 2. The extraction yield was 9.5% for the pectic polysaccharides extracted with HCl (PP-I), 1.8% for the pectic polysaccharides (PP-II) obtained using citric acid, and 7.2% for the Ca-bound pectic polysaccharides-enriched fraction (CaPP-II) from the additional extraction using ammonium oxalate. Extraction using the stronger mineral acid (Method I) produced a yield comparable to that obtained with the milder organic acid, in conjunction with the chelating agent (Method II + ammonium oxalate). Statistical analysis confirmed that there were no significant differences in extraction yields among these methods (*p* > 0.05).

However, yield alone is not a sufficient indicator of extraction efficiency, and the monomeric composition of the fractions (Table 3) and their molecular weight (M_w_) distribution must also be considered to assess their structural features and potential functionality, as strong mineral acids may compromise polysaccharide integrity through uncontrolled depolymerization and reduction of molecular weight. Monomer composition analysis (Table 3) revealed that all obtained potential pectic polysaccharides-enriched fractions contained relatively low levels of the significant component for pectin—GalA (PP-I (15.9%), PP-II (22.7%), and CaPP-II (10%)). Since a typical pectin polysaccharide should comprise at least 65% GalA, these fractions cannot be classified as pectins (E-440) [27]. Given that the obtained fractions contained Ara as the most abundant monomer (40–45%), as well as 12–15% Gal, and 5–13% Xyl, it is evident that co-extraction of polysaccharides other than pectin took place. Consequently, these fractions can be classified as a mixture of different polysaccharide types, largely comprising arabinose-rich polysaccharide mixtures, such as arabinans and arabinogalactans, branches of RG-I, but also xyloglucan, a type of hemicellulosic polysaccharide [28]. Statistical analysis also confirmed that there is no significant difference in Ara content among the PP-I, PP-II, and CaPP-II fractions.

For example, previously published data on *B. campestris* report the extraction of arabinan and acidic arabinogalactans following hot acidic water treatment [29]. Previous studies have also shown that arabinogalactans, such as those isolated from RSM, could exhibit notable pharmacological activities, including anti-allergic, immunomodulatory, and anti-inflammatory effects [30].

It is also noteworthy that the obtained polysaccharide-enriched fractions (PP-I, PP-II, and Ca-PP-II) contained Ara as the most abundant monomer (40–45%), while the significant monomer for pectic polysaccharides—GalA was present at only up to 22%. Additionally, these fractions contained between 10 and 35% protein of dry matter (Supplementary Information File Table S1). These results collectively indicate low selectivity of the applied acid extraction procedures under these experimental conditions, leading to lower purity of the carbohydrate fractions.

#### 3.1.2. Enzyme-Assisted Extraction

Both acid extraction methods exhibit notable limitations when the goal is to obtain a pectic polysaccharide-enriched fraction for subsequent POS production. These limitations include insufficient protein removal and a substantial content of monosaccharaides originating from non-pectic polysaccharides in the precipitates obtained after fractionation. According to literature, rapeseed meal (RSM) cell walls comprise a highly integrated polysaccharide network, including homogalacturonan, rhamnogalacturonan, arabinan, and arabinogalactan, closely associated with cellulose, xyloglucans, and xylans through predominantly non-covalent (hydrogen-bonding and electrostatic interactions) and occasional covalent interactions (e.g., Ca^2+^ cross-linking in homogalacturonan regions) [29,31]. Proteins are mainly localized within intracellular protein bodies enclosed by this rigid matrix. Given this structural complexity, enzyme-assisted extraction (EAE) of pectic polysaccharides can be tailored to preserve polymer integrity or promote depolymerization [32]. By applying enzymes with cellulolytic and hemicellulolytic activities, they will hydrolyze *β*-1,4-glycosidic bonds in cellulose and hemicellulose, weakening the cell wall framework, increasing porosity, and reducing pectin entrapment [33]. Concurrently, pectinolytic enzymes, when present, cleave the homogalacturonan backbone, lowering the degree of polymerization and enhancing solubility. In general, enzymatic action may also disrupt linkages between pectin and hemicellulose (e.g., rhamnogalacturonan–arabinoxylan associations), further facilitating its release.

Therefore, this study hypothesizes that EAE using a Cellic^®^ CTec3 HS preparation, rich in cellulolytic and hemicellulolytic activities, will alter the cell wall matrix surrounding pectin and enhance its extractability, but will not disrupt its structure. Furthermore, pretreatment involving phenolic removal followed by proteolysis may improve pectin release by disrupting protein–polysaccharide interactions. Hence, Method III was developed (Figure 1), comprising a two-step EAE process employing the commercial enzyme preparations Alcalase^®^ 2.4 L (Alcalase^®^) and Cellic^®^ CTec3 HS (Cellic^®^) (Figure 1).

The experimental conditions for deproteinization with Alcalase^®^ (a commercial protease preparation) were reported in our earlier study [16], where it was demonstrated to efficiently remove proteins and enhance the purity of the obtained xylan fraction. In this study, Alcalase^®^ was applied to remove the majority of cell-wall-associated proteins from alcohol-insoluble residue of RSM (AIR-RSM, Figure 1), yielding deproteinized RSM (DP-RSM, Figure 1). This step also produced a protein-rich fraction (Figure 1), which comprises 69% of AIR-RSM protein content, indicating efficient protein extraction and improved accessibility of the cell wall matrix for subsequent pectic polysaccharides recovery [19,34,35]. Notably, isolated rapeseed protein has been authorised as a novel food by the EFSA NDA Panel [36,37], which highlights the added value of this fraction and further supports the comprehensive valorisation of RSM achieved in our study.

The next step involved treating the resulting DP-RSM with Cellic^®^, an enzyme complex that contains cell wall-degrading enzymes with cellulase and hemicellulase activities, to remove non-pectic carbohydrates in the soluble fraction. Based on the literature, DP-RSM treatment with Cellic^®^ preparation was chosen to be performed in Na-citrate buffer pH 5.0, at a solid-liquid ratio of 1:10 (*w*/*v*) and 40 °C with an enzyme concentration of 1% (*v*/*v*) [38,39]. The incubation time was varied (8 h and 24 h) to obtain a more efficient isolation method. The effects of Cellic^®^ preparation on pectic polysaccharides isolation were evaluated by determination of monomer composition by GC-FID (Table 3), M_w_ distribution by HPSEC-ELSD (Figure 2), and FTIR analysis (Supplementary Information file Figure S1) of polysaccharide precipitate (PP-EAE, Figure 1) to elucidate the relative significance of this treatment. Furthermore, the enzymatic approach aimed to address the limitations of acid extraction by providing a more selective and environmentally sustainable method for pectic polysaccharides recovery.

The enzymatic treatment of deproteinized meal (DP-RSM) for 24 h resulted in a pectic polysaccharides-enriched fraction (PP-EAE(24 h), Table 3) containing 45.4% GalA as the dominant monomer. The Ca-bound pectic polysaccharides-enriched fraction (CaPP-EAE(24 h), Figure 1), obtained after additional ammonium oxalate extraction of the residual material, exhibited an even higher GalA content of 56.8%. In contrast, acid-extracted fractions (PP-I, PP-II, and CaPP-II) primarily contained Ara as the most abundant monomer. The protein content of the EAE extracted fractions was approximately 4% (Supplementary Material, Table S1), indicating that this method yields purer pectic fractions compared to the applied acid extraction techniques. These findings demonstrate that the applied two-step EAE selectively isolates GalA-rich pectic polysaccharides, likely due to the removal of proteins *via* Alcalase^®^ treatment and the specificity of Cellic^®^ enzymes for cellulosic and hemicellulosic components, thereby contributing to the preservation of the native structural features of pectic polysaccharides and yielding fractions of higher purity. Conversely, acid extraction at experimental conditions used in this study, although less selective, proved to be more efficient in terms of overall yield (9.52% for HCl extraction and 7.24% for citric acid-treated RSM), producing a broader spectrum of arabinose-rich polysaccharides from RSM.

Therefore, while acid extraction can achieve higher yields under certain conditions, EAE treatment offers significant advantages in selectivity, structural integrity, and protein removal, positioning it as a valuable complementary approach for the targeted recovery of high-purity GalA-rich pectic polysaccharides.

The GalA content determined for both EAE fractions was comparable to values reported for pectins isolated from okra (46–56%) [40], sugar beet (29–52%) [41], and pomegranate peel (19.9–30.8%) [39]. In contrast, polysaccharides isolated from rapeseed husk and kernel have been reported to display lower heterogeneity, consisting predominantly of Ara, Gal, Glc, and Xyl, with GalA representing up to only 10% of the total monosaccharide composition [42].

Despite the moderate content of GalA (approximately around 50%), the simultaneous enrichment in Ara, Gal, and Rha is consistent with the structural fingerprint of RG-I domains from rapeseed [25]. It should be noted that in terms of monomeric composition, shorter incubation times (8 h) once more proved to be insufficient for efficient pectic polysaccharides extraction, as the GalA content in both obtained pectic polysaccharides-enriched fractions (PP-EAE(8 h) and CaPP-EAE(8 h)) was approximately half that observed after 24 h, accompanied by a high proportion of Ara (up to 30%).

M_w_ distribution analysis revealed distinct profiles between the 8-h and 24-h fractions (Figure 2). The PP-EAE(8 h) predominantly contained a single M_w_ fragment with an average M_w_ of 12.2 kDa, accounting for 83.2% of the fraction, along with two minor peaks at approximately 0.7 kDa (12.9%) and 64.5 kDa (3.8%). This suggests that, after 8 h of enzymatic treatment, oligosaccharides are the main products isolated. Extending the enzymatic treatment to 24 h significantly influenced the M_w_ profile of the extracted fractions. The PP-EAE(24 h) fraction exhibited two predominant peaks at average M_w_ of 19.9 kDa and 43.4 kDa, accounting for 61.2% and 36.3% of the total fraction, respectively, indicative shift of the most abundant peaks towards significantly higher degrees of polymerization. Additionally, the appearance of a high-molecular-weight fragment (>416 kDa), representing 3.5% of the PP-EAE(24 h), indicates significant extraction of the polysaccharide fraction after prolonged enzymatic treatment. Notably, the extraction yield for PP-EAE after 8 h was approximately half that obtained after 24 h (3.2% versus 6.8%), and only 0.5% of the CaPP-EAE(8 h) was recovered. These findings suggest that an extraction duration of 8 h is insufficient for effective polysaccharide extraction from RSM using Cellic^®^ preparation, whereas extending the process to 24 h enhances polysaccharide yield and shifts the M_w_ distribution towards larger, more complex structures. The statistical analysis confirmed this conclusion, since portions of the fragment with an average M_w_ between 5 and 20 kDa within fraction show a significant difference between fractions PP-EAE(8 h) and PP-EAE(24 h). Additionally, PP-EAE(24 h) contains a 20 ˂ M_w_ ˂ 50 kD fragment which PP-EAE(8 h) does not possess.

Moreover, the Ca-bound pectic polysaccharides obtained by ammonium oxalate extraction after 24 h of Cellic^®^ treatment (CaPP-EAE(24 h) fraction) displayed 4 average M_w_ peaks at 0.04 kDa, 1.48 kDa, 17.1 kDa and 451.8 kDa, representing 1.6%, 13.0%, 51.7% and 34.0% of the fraction, respectively, confirming it is long-chain oligosaccharides (degree of polymerization (DP) ˃ 20) and polysaccharides. Additionally, these findings align with numerous studies indicating that pectic polysaccharides isolated with chelating agents from fruits such as citrus, berries, mango, and banana possess higher M_w_ compared to those obtained through acid extraction [43,44]. The extraction yields after 24 h were 6.8% for PP-EAE(24 h) and 2.7% for CaPP-EAE(24 h), indicating the efficacy of enzymatic treatment in producing pectic polysaccharides-enriched fractions

Several studies have reported the isolation of pectins from rapeseed (*B. napus*) residues generated during the production of rapeseed oil or biodiesel, as well as the characterization of cell wall polysaccharides present in *B. napus* [11,29,45]. For instance, Jeong et al. [11] employed an EAE approach, utilizing a synergistic treatment with Celluclast^®^ (a commercial cellulase) and Alcalase^®^ (a protease) over 270 min to extract pectic polysaccharides from rapeseed cake (RSC). They observed a GalA content of 64.5% in the isolated pectin fraction, slightly higher than the 56.8% obtained in the present study. This variation may be attributed to differences in raw material sources, enzyme preparations used, and extraction conditions, as the results are specific to the rapeseed raw materials employed (RSM or RSC). In general, further optimization of the extraction parameters would be necessary to adapt the process for other rapeseed species or RSM production conditions. Another investigation examined the relationship between extraction methods and the structure of pectic polysaccharide fractions from RSM. Pustjens et al. [29] obtained multiple polysaccharide fractions with GalA contents ranging from 5% to 71% *via* sequential isolation techniques, including ethanol extraction, chelation with CDTA, and high ionic strength alkali solutions (NaOH, KOH). These findings align with those of Eriksson et al. [45], who reported pectic polysaccharides with uronic acid contents up to 52% following a three-step extraction process involving ethanol, pH-adjusted CDTA, and sodium carbonate. Collectively, these studies underscore the significant influence of extraction methodology on GalA content, with chelating agents and enzymatic treatments generally facilitating the extraction of pectic polysaccharides with higher uronic (GalA) acid content.

Our results are consistent with these prior findings, demonstrating that enzymatic and chelating agent treatments effectively enrich pectic polysaccharide isolation from RSM. Specifically, it was observed extraction yields of 6.8% for PP-EAE(24 h) and 2.7% for CaPP-EAE(24 h), comparable to 6.85% with Celluclast^®^-Alcalase^®^ [11] and 5.2% with CDTA at pH 6.5 (0.02 M) [45].

Regarding molecular weight (M_w_) distribution, our extracts ranged from 440 to 25 kDa, aligning with previous reports indicating that RG-I–enriched pectins in Brassicaceae species are often highly branched, with structures rich in arabinan and galactan domains rather than homogalacturonan (HG). This structural feature has been associated with higher Mw fractions, particularly when extracted using chelating agents, as observed in study of Pustjens et al. [29].

Based on the comprehensive analysis of the presented results (Figure 2 and Table 3), an incubation time of 24 h was determined to be the best for treatment with the Cellic^®^ preparation. Therefore, further structural analysis and evaluation of pectic polysaccharides fractions will be performed only on the two fractions obtained after 24 h of treatment with the Cellic^®^ preparation and additional extraction with ammonium oxalate.

**Structural characterization of pectic polysaccharides fractions obtained using enzyme-assisted methodology.**

Regarding the structural characteristics of polysaccharides in fractions isolated in different stages of EAE, notable differences were observed (Table 4).

The pectic polysaccharide-enriched fraction obtained after 24 h of enzymatic treatment with Cellic^®^ (PP-EAE(24 h)) contains approximately 36% HG. In contrast, the relatively high Rha content (9.1%) indicates that RG-I is the dominant structural domain, making up about 48% of the total pectic polysaccharides (Table 4). Considering that the original material (AIR-RSM) comprised roughly 69% RG-I within its total polysaccharides, these findings suggest that a significant portion of RG-I polysaccharides was successfully extracted and recovered in this fraction. The low degree of branching (DB) and extent of branching (EB) of RG-I (Table 4) imply that this fraction has relatively few side chains, indicating a less branched (“smooth”) pectic structure.

The high levels of Gal (12.5%), Ara (17.6%), and Xyl (7.6%), combined with a relatively low GalA (45.0%) content, support the presence of substantial RG-I domains. According to the literature in rapeseed (*B. napus*) cell walls, RG-I side chains are typically composed of *α*-(1→5)-linked L-arabinan branched at O-2 position, *β*-(1→4)-linked D-galactan, and *β*-(1→3)-linked D-galactan at O-6 with galactan or arabinogalactan type II moieties [29]. The presence of co-extracted hemicellulosic polysaccharides, such as xyloglucans (XXGG-type and XXXG-type) and glucuronoxylans, is also evident and in accordance with the literature [29]. Moreover, previous studies have shown that pectin extracted from rapeseed (*B. napus*) is particularly rich in xylogalacturonan, although its relative abundance depends heavily on the extraction and isolation methods used [29]. The pectic polysaccharides in the cell walls of *B. campestris* seed cake, a major oilseed and vegetable crop closely related to *B. napus* within the Brassicaceae family, have been found to share similar structural characteristics [24].

On the other hand, the Ca-bound pectic polysaccharides fraction (CaPP-EAE(24 h)) predominantly consists of HG, accounting for approximately 53%. This HG content is higher than that reported for pectins derived from various plant sources, such as okra (HG ≤ 45%) [46], and is comparable to pectins extracted from soybean [47] and green tea leaves [48]. However, it is lower than the HG content observed in blackcurrant (66–77%) [43] and apple and citrus pectins (approximately 71–78%) [49]. Additionally, pectin isolated from rapeseed hulls by extraction with aqueous ammonium oxalate contained residues mainly of GalA (76%) and rhamnose (2–3%), indicating that HG is the predominant polysaccharide type in this agro-waste [50].

Considering the Rha content (3.6%) and the structural characteristics of RG-I, reflected by a high degree of branching (DB = 15.77) and extent of branching (EB = 6.38) (Table 4), together with elevated Ara (13.20%) and Gal, 9.80% levels, it can be concluded that the Ca-bound pectic polysaccharide fraction is enriched in highly branched pectic domains. These include arabinan, galactan, and arabinogalactan side chains linked to the RG-I backbone, as previously stated [51], as well as HG regions that may undergo structural modifications. Specifically, HG can be *β*-xylosylated at the O-3 position or apiosylated at the O-2 and/or O-3 positions, giving rise to xylogalacturonan and apiogalacturonan, respectively [51]. Such features, together with the presence of complex galacturonan domains, indicate a structurally diverse and extensively substituted pectic network within the Ca-bound fraction.

Based on the established knowledge of the carbohydrate composition of RSM cell walls [29], and in accordance with the data presented in Table 3 and Table 4, it is evident that the pectic polysaccharides-enriched fraction contains a significant proportion of hemicellulose and cellulose microfibrils closely associated with pectin. This suggests that the cell wall polysaccharide matrix of RSM is highly interconnected. A similar conclusion has been reported in a study by Ralet et al. [41].

Moreover, the two pectic polysaccharides fractions obtained from deproteinized rapeseed meal after 24 h of EAE (PP-EAE) and additional extraction using ammonium oxalate (CaPP-EAE), displayed the most pronounced structural changes among all samples. These modifications are consisting with extensive removal of neutral sugars and non-pectic polysaccharides. Both fractions showed a clear enrichment in pectic components. This enrichment was accompanied by a substantial increase in HG content (from 4.9% in AIR-RSM), reaching 36.3% in PP-EAE and a higher 53.2% in CaPP-EAE, which suggests selective hydrolysis or solubilization of RG-I side chains and hemicellulosic residues. Collectively, these data demonstrate that 24 h of EAE promotes the recovery of structurally simplified, highly HG-enriched pectins while substantially reducing non-pectic carbohydrates.

In addition, FTIR spectroscopy was employed in this study for the determination of the degree of methyl esterification of the fractions obtained after 24 h of EAE and additional extraction using ammonium oxalate. The obtained FTIR spectra are presented in Figure S1. In summary, both fractions exhibited characteristic absorption bands around 1740 cm^−1^, 1640 cm^−1^, and 1420 cm^−1^, which are attributed to the C=O stretching vibration of methyl esterified carboxyl groups, carboxylate anion (COO-) stretching vibration, and to the C=O stretching vibration of free carboxyl groups, respectively [52]. However, the FTIR spectra of both fractions showed only a very weak band at 1740 cm^−1^, meaning that esterified carboxyl groups are largely absent. This indicates that both extracted pectic polysaccharides fractions possess a low degree of methyl esterification (DM < 10) [53]. The significantly lower DM resulting from the enzymatic extraction at longer times (24 h) and at relatively high temperatures (50 °C), and even to a possibly better extraction of more strongly bound pectic polysaccharides, possibly of lower DM. Similar results regarding the absence of methyl esterified carboxyl groups in Ca-bound pectins isolated using chelating agents from different plant materials were confirmed in the literature [43,54]. Based on FTIR analysis, it can be concluded that the PP-EAE and CaPP-EAE fractions are classified as low-methoxyl pectic polysaccharides, with a DM below 50% [55].

Based on the obtained results, the two-step enzymatic extraction using the protease preparation Alcalase^®^, followed by the cellulase/hemicellulase preparation Cellic^®^ over 24 h, to first obtain the PP-EAE fraction and then, after an additional ammonium oxalate extraction, the CaPP-EAE fraction, appears to be the most effective approach among the conditions tested in this study for isolating pectic polysaccharides from RSM. Notably, the CaPP-EAE fraction was structurally characterized as an LM pectic polysaccharide with a high content of HG regions and long-chain RG-I domains with substantial branching, and the molecular weight (M_w_ > 50 kDa), indicating that a significant portion of its macromolecular size was retained under these extraction conditions. Although neutral sugars such as mannose, glucose, and xylose remain present, the overall composition shows a clear enrichment in pectic structural features relative to the starting material. This structural definition and the preservation of sizeable polymeric domains suggest that this fraction could be suitable for subsequent controlled enzymatic depolymerization aimed at generating structurally diverse POS with potential prebiotic functionality. However, further research is necessary to confirm the applicability of this fraction for such purposes and to explore the functional properties of the resulting oligosaccharides.

### 3.2. Enzymatic Production of Pectic Oligosaccharides from Isolated Fraction

POS are non-digestible oligosaccharides that have the potential to exhibit prebiotic activity, primarily through the selective stimulation of beneficial intestinal microbiota, such as *Bifidobacteria* and *Lactobacilli* in the colon. The enzymatic production of POS has been extensively explored due to the high specificity and selectivity of the enzymes involved, which also confer safety advantages by minimizing undesirable chemical modifications compared to other methods.

**Figure 3:**
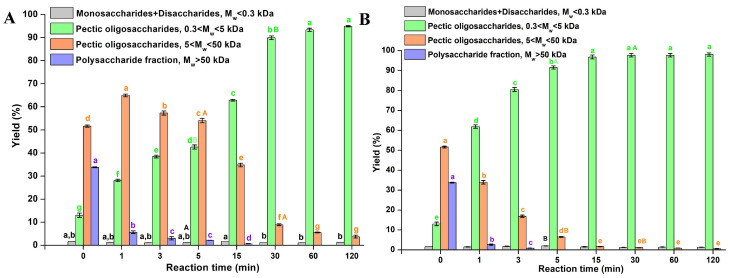
Hydrolysis of CaPP-EAE fraction with Pectinex^®^. (**A**) Reaction performed with enzyme concentration of 0.5% (*v*/*v*); (**B**) Reaction performed with enzyme concentration of 1% (*v*/*v*). Different characters (a–g) on the graphs indicate a statistically significant difference (*p* < 0.05) between the yields of each fragment (M_w_ ˂ 0.3 kDa; 0.3 ˂ M_w_ ˂ 5 kDa; 5 ˂ M_w_ ˂ 50 kDa; and M_w_ ˃ 50 kDa) of CaPP-EAE fraction across different reaction times. Different characters (A–B) within the same fraction fragment at 5 and 30 min indicate a statistically significant difference (*p* < 0.05) between those time points for each enzyme concentration (0.5% and 1% (*v*/*v*)).

In this study, the enzyme preparation Pectinex^®^ Ultra Passover (Pectinex^®^) was utilized to generate POS from the CaPP-EAE(24 h) fraction. Pectinex^®^ is a complex enzyme formulation containing pectinases (polygalacturonase, 5000 PGNU/g), hemicellulases (such as endo-1,4-*β*-xylanase,), and *β*-glucanase from *Aspergillus aculeatus* [56]. Two enzyme concentrations (0.5 and 1% (*v*/*v*), 125 and 250 µL/g CaPP-EAE(24 h)) were tested to evaluate their efficacy, to maximize oligosaccharide yield while minimizing monosaccharide production. The hydrolysis POS yield profiles are shown in Figure 3.

After only 1 min of reaction, the commercial pectinase preparation generated a mixture of long-chain POS of average M_w_ of 17 kDa and short-chain POS of average M_w_ of 2 kDa from the polysaccharide fraction of M_w_ 452 kDa and the long-chain oligosaccharide fraction of M_w_ 17.1 present in CaPP-EAE, demonstrating the feasibility of enzymatically catalyzed POS production. The POS profile observed at the onset of CaPP-EAE degradation by the Pectinex^®^ preparation suggests the presence of polygalacturonase activities capable of generating a mixture of oligomers of varying sizes, which is consistent with enzymes displaying predominantly endo-acting behavior. The initial appearance of long-chain POS followed by shorter oligomers may indicate a progressive depolymerization process. Additionally, the low monosaccharide yield could reflect limited exo-polygalacturonase activity.

When the reaction was performed with 1% (*v*/*v*) enzyme, an almost complete conversion of the polysaccharide fraction of M_w_ 452 and long-chain oligosaccharide fraction of M_w_ 17.1 present in CAPF-EAE into short-chain POS of M_w_ 2 kDa was achieved within 5 min. In addition, there was no significant difference in short-chain POS yield after this reaction time. The short-chain polysaccharide of 2 kDa corresponds to potential DP values between 10 and 11. Under the 0.5% (*v*/*v*) enzyme condition, the maximum short-chain POS yield was achieved after 30 min, with statistical analysis showing no significant difference in yield between samples at 60 and 120 min. Comparing the yields of short- and long-chain POS at enzyme concentrations of 0.5% and 1% (*v*/*v*) for reaction times of 5 and 30 min, it is evident that the composition of the obtained fractions after hydrolysis with Pectinex^®^ differs significantly.

The high hydrolytic efficiency of this preparation toward the CaPP-EAE fraction likely stems from the preference of endo-polygalacturonases for low-esterified substrates [57,58]. This suggests that the enzymatic tailoring of pure polygalacturonic acid and that of crude pectic polysaccharide extracts from RSM may exhibit qualitatively similar degradation patterns, although further confirmation is needed to substantiate this comparison [59]. The use of pectinolytic enzyme preparations for the production of POS from agro-industry by-products has been reported. For example, pectinolytic enzymes were used to obtain a mixture of oligomers of M_w_ between 1.4 and 1.7 kDa from bergamot peel [10] and sugar beet pulp [60]. However, it should be noted that many pectinolytic enzyme preparations used for POS production from fruits or agro-industrial by-products typically generate substantial amounts of monosaccharides, requiring additional purification of the resulting POS fractions. In contrast, the POS obtained with the Pectinex^®^ preparation in this study showed comparatively lower monosaccharide levels.

The biological activity of oligomers such as the POS fraction obtained in this study with 0.5% (*v*/*v*) of Pectinex^®^ preparation over 30 min is modulated by factors including M_w_ distribution, degree of esterification, and compositional characteristics. Consequently, to evaluate the prebiotic potential of the obtained POS fraction derived from RSM, in vitro tests were conducted with single-strain cultures.

### 3.3. Preliminary Evaluation of Prebiotic Potential of Obtained Pectic Oligosaccharides from RSM

**Figure 4:**
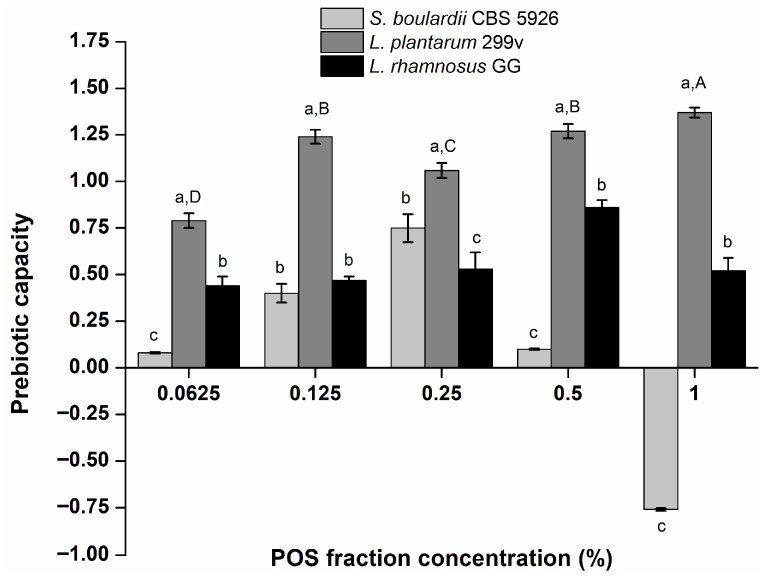
The prebiotic capacity of various POS fraction concentrations. Data are presented as mean value ± standard deviation. Different lowercase letters (a, b, c) above the bars at each tested POS fraction concentration indicate statistically significant differences in prebiotic capacity between the individual gut microbes. Different uppercase letters (A, B, C, D) indicate statistically significant differences in prebiotic capacity of *L. plantarum* between the tested POS fraction concentration (*p* < 0.05, one-way ANOVA with Tukey’s post-hoc test).

Prebiotic potential screening of the POS mixture obtained from CaPP-EAE(24 h) fraction using 0.5% (*v*/*v*) Pectinex^®^ preparation for 30 min, was evaluated by examining its effect on the in vitro growth in single-strain models using selected probiotic bacterial and yeast strains such as such as *L. plantarum* 299v, *L. rhamnosus* GG, and *S. boulardii* CBS 5926, as well as an indicator strain of a potentially pathogenic bacteria *E. coli* ATCC 25922. The effect of different POS fraction concentration on the growth (CFU/mL) of selected probiotic bacterial and yeast strains, after 24 h of incubation was presented on Figure S2 (Supplementary Information file). A simplified screening assessment was conducted by calculating the prebiotic capacity (PC) after 24 h (Equation (1)), as shown in Figure 4.

Considering that PC values greater than 0 indicate a selective growth stimulation of selected probiotic strains relative to *E. coli* in the presence of the tested sample, Figure 4 demonstrates that the most pronounced and statistically significant growth stimulation (*p* < 0.05) due to POS supplementation was observed for *L. plantarum* 299v within the entire examined concentration range (0.0625–1%) in comparison with other tested strains. Among all tested strains, *L. plantarum* 299v consistently exhibited the highest PC values, indicating a stronger relative growth response to POS supplementation under the applied in vitro conditions. The highest (PC value 1.38) and statistically significant prebiotic activity (*p* < 0.05) was observed at a concentration of 1% POS, compared to all lower concentrations tested, suggesting a concentration-dependent enhancement of selective substrate utilization by this strain. The effect of different POS fraction concentrations on the growth (CFU/mL) of *L. plantarum* 299v, *L. rhamnosus* GG, and *S. boulardii* CBS 5926, as well of *E. coli* ATCC 25922, after 24 h of incubation also confirmed the same trend as observed in the case of PC.

In contrast, even though *L. rhamnosus GG* demonstrated a positive growth response, its PC values did not increase to the same extent at higher concentrations (the highest PC value was observed at 0.5% POS), indicating potential differences in substrate affinity or metabolic efficiency between the two Lactobacillus strains. Finally, these high POS concentrations led to a decrease of PC values calculated for *S. boulardii*. Even a negative PC value was observed for *S. boulardii* at the highest concentration, suggesting that the tested compound favored the growth of the pathogenic strain over the probiotic strain under the examined conditions. This result does not necessarily imply preferential stimulation of the pathogenic strain, but it rather reflects differential metabolic capacity or environmental tolerance among the tested microorganisms. Namely, it should be noted that the release of GalA during POS mixture utilization by microorganisms may impact medium pH and thereby influence growth responses, particularly at higher POS concentrations. Concerning bacteria from the family *Lactobacillaceae*, the results obtained in this study are in accordance with previous research, which also reported the stimulation of different strains of lactic acid bacteria by the application of structurally different POS from various sources [10,61,62].

In particular, for the bacterial species *L. rhamnosus*, other authors reported that the application of citrus POS stimulated the growth of the bacterial strain *L. rhamnosus* CRL1505 [61]. These results indicate that strains within the same species may possess similar metabolic pathways towards the utilization of POS. According to Mitterdorfer et al. [63], who examined the possibility of using a wide range of carbohydrates, indicated a high affinity of different yeast strains to the use of compounds based on glucose and fructose, specifically FOS. They also reported the inability of all tested strains to metabolize rhamnose, which may be part of the structure of the tested POS, which could potentially be an explanation for the results shown in this study.

These findings indicate a higher prebiotic potential of the POS fraction obtained from RSM for the tested strains from the *Lactobacillaceae* family, compared to the yeast strain. Nevertheless, it should be noted that the obtained results are based on a single-strain model, which allows for a controlled and targeted assessment of the response of selected microorganisms and initial evaluation of prebiotic potential, but does not fully reflect the complexity of the gut microbiota. To address this limitation, future studies should employ fecal inoculum-based models that better reflect a diverse microbial community and allow monitoring of change in microbial composition and metabolite production. Such approaches, along with clinical studies, would enable a more comprehensive assessment of the prebiotic effect.

## 4. Conclusions

In conclusion, this study presents a promising laboratory-scale approach for the production of pectic polysaccharides from rapeseed meal (RSM). Pectic polysaccharides were obtained *via* a two-step enzyme-assisted extraction (EAE) process using Alcalase^®^ and Cellic^®^ preparations, followed by ammonium oxalate extraction. Under the applied conditions, the EAE strategy demonstrated improved selectivity and protein removal compared to the conventional acid extraction methods evaluated in this study, enabling the recovery of structurally defined pectic polysaccharide fractions. The resulting Ca-bound polysaccharide-enriched fraction (CaPP-EAE) was structurally characterized, revealing two M_w_ populations (M_w_ = 17.1 kDa and 452 kDa) indicative of long-chain oligosaccharides and polysaccharides. Also, it was demonstrated that this fraction contains a high galacturonic acid content (56.8%), a low degree of methylation (10%), and the presence of both homogalacturonan (HG) and rhamnogalacturonan-I (RG-I) domains with side chains. Subsequent enzymatic treatment with Pectinex^®^ Ultra Passover converted detected fragments into a mixture enriched in short-chain pectic oligosaccharides (POSs), with a low monosaccharide content (4.3%), indicating limited exo-glycosidase activity under the applied conditions. Further on, the simplified in vitro screening method demonstrated a selective growth response of *Lactobacillaceae* strains compared to the yeast strain under the tested conditions, suggesting the putative prebiotic potential of the obtained POS fraction.

Although the proposed approach demonstrates potential at the laboratory scale, several factors, including enzyme costs, processing time (~24 h), and variability of RSM feedstock, may affect scalability and industrial implementation. Consequently, further work addressing process optimization, economic feasibility, and scale-up is necessary before practical application can be considered. Overall, this study provides a solid foundation for the further development of strategies aimed at producing structurally defined pectic oligosaccharides from RSM and supports future investigations into their functionality as emerging prebiotic ingredients in more physiologically relevant systems.

## Figures and Tables

**Table 1:** Monomeric composition of AIR-RSM polysaccharides (% total identified monosaccharides *). All data are presented as mean value ± standard deviation.

Species	Man	Rha	GalA	Glc	Xyl	Gal	Ara
AIR-RSM	1.7 ± 0.1	4.8 ± 0.5	9.7 ± 0.9	8.2 ± 0.4	15.7 ± 1.2	17.1 ± 0.9	42.8 ± 2.8

* Expressed as anhydro-monosaccharide units: Man—mannose; Rha—rhamnose; GalA—galacturonic acid; Glc—glucose; Xyl—xylose; Gal—galactose; Ara—arabinose.

**Table 2:** Pectic polysaccharides extraction yields on a mass basis. All data are presented as mean value ± standard deviation. Different characters (a, b, and c) indicate statistically significant differences at *p* < 0.05 in the extraction yields of the obtained pectic polysaccharide fractions using different extraction methods.

Polysaccharides Fractions	Method	Extraction Yield, %
**Pectic polysaccharides fraction I (PP-I)**	Method I—HCl	9.52 ± 1.32 ^a^
**Pectic polysaccharides fraction II (PP-II)**	Method II—Citric acid	1.84 ± 0.42 ^c^
**Ca-bound pectic polysaccharides fraction (CaPP-II)**	Method II—citric acid + ammonium oxalate	7.24 ± 0.97 ^a,b^
**Pectic polysaccharides fraction EAE** **(PP-EAE)**	Enzyme-assisted fractionation (EAE)	6.80 ± 0.74 ^b^
**Ca-bound pectic polysaccharides fraction EAE** **(CaPP-EAE)**	Enzyme-assisted fractionation (EAE) + ammonium oxalate	2.72 ±0.91 ^c^

**Table 3:** Monomeric composition of obtained pectic polysaccharides fractions (% total identified monosaccharides *). All data are presented as mean value ± standard deviation. Different characters (a, b, c, d, e, and f) in the same column signify a statistically significant difference at *p* < 0.05 between species.

Species	Man	Rha	GalA	Glc	Xyl	Gal	Ara
PP-I	2.68	9.30	15.89	10.69	5.34	15.62	40.47
	±0.99 ^c, d^	±0.25 ^a^	±1.80 ^e,f^	±0.98 ^a^	± 1.58 ^d^	±1.42 ^a,b,c,d^	±3.69 ^a^
PP-II	2.03	4.38	22.73	6.83	10.56	12.10	41.38
	±012 ^d^	±0.56 ^b^	±1.54 ^c,d^	±1.54 ^b,c^	±1.24 ^a,b^	±1.20 ^d,e^	±1.56 ^a^
CaPP-II	11.27	4.11	9.99	7.16	7.11	14.46	45.90
	±0.52 ^a^	±0.87 ^b^	±1.74 ^f^	±0.73 ^b^	±1.45 ^c,b,d^	±2.14 ^a,b,c,d^	±1.78 ^a^
PP-EAE(8 h)	7.00	5.40	20.40	4.90	13.30	18.60	30.50
	±0.84 ^b^	±1.25 ^b^	±1.45 ^d,e^	±0.25 ^c,d^	±2.12 ^a^	±2.46 ^a^	±2.45 ^b^
CaPP-EAE(8 h)	10.40	3.80	27.00	11.80	10.10	13.20	23.80
	±1.23 ^a^	±0.75 ^b^	±2.14 ^c^	±0.43 ^a^	±2.47 ^a,b^	±0.98 ^b,c,d,e^	±2.14 ^c^
PP-EAE(24 h)	3.50	9.10	45.40	4.30	7.60	12.50	17.60
	±0.69 ^c,d^	±0.89 ^a^	±2.45 ^b^	±0.57 ^c,d^	±0.85 ^b,d^	±1.24 ^c,d,e^	±1.24 ^d^
CaPP-EAE(24 h)	4.90	3.60	56.80	3.40	8.80	9.80	13.20
	±1.23 ^b,c^	±0.54 ^b^	±4.15 ^a^	±0.15 ^d^	±1.45 ^a,b,d^	±1.45 ^e^	±1.45 ^d^

* Expressed as anhydro-monosaccharide units: Man—mannose; Rha—rhamnose; GalA—galacturonic acid; Glc—glucose; Xyl—xylose; Gal—galactose; Ara—arabinose.

**Table 4:** Structural parameters based on the monomeric composition of AIR-RSM, pectic polysaccharides, and Ca-bound pectic polysaccharides-enriched fractions obtained using EAE with Cellic^®^ for 24 h and additional extraction using ammonium oxalate.

Species	HG (%)	RG-I Content (%)	DB of RG-I *	EB of RG-I *	LP *	PP *
PP-EAE(24 h)	36.3	48.3	4.98	3.31	1.16	10.85
CaPP-EAE(24 h)	53.2	30.2	15.77	6.38	2.13	10
AIR-RSM	4.9	69.5	2	12.4	0.14	8.02

* Degree of branching of RG-I (DB of RG-I); Extent of branching of RG-I (EB of RG-I); the linearity of pectin backbone (LP); the purity of obtained pectin (PP) (Supplementary Information file).

## Data Availability

The original contributions presented in the study are included in the article/Supplementary Materials; further inquiries can be directed to the corresponding author.

## Supplementary Materials

The following supporting information can be downloaded at: https://www.mdpi.com/article/10.3390/foods15081338/s1. Figure S1: FTIR spectra of the obtained pectic polysaccharide-enriched fractions; Figure S2: Effect of different POS fraction concentration on the growth of: (a) *Saccharomyces boulardi* CBS 5926, (b) *Lactiplantibacillus plantarum* 299v, (c) *Lacticaseibacillus rhamnosus* GG, and (d) Escherichia coli ATCC 25922, after 24 h of incubation; Table S1: Protein content. References [53,55] are cited in the Supplementary Materials.
