# Supplementary material for: Pectic Polysaccharides Recovery from Rapeseed Meal via Conventional and Enzyme-Assisted Extraction Techniques: Toward Emerging Prebiotic Pectic Oligosaccharide Development
Source: Foods. 2026 Apr 12;15(8):1338. doi: 10.3390/foods15081338 (PMC13114823; doi:10.3390/foods15081338)
Supplement: Supplementary file 1 [file foods-15-01338-s001.zip › foods-4224152-supplementary.pdf]

SUPPLEMENTARY FILE

for

Pectic Polysaccharides Recovery from Rapeseed Meal *via* Conventional and Enzyme-assisted Extraction Techniques: Toward Emerging Prebiotic Pectic Oligosaccharide Development

Katarina Banjanac[1], Milica Veljković[1,*], Milica Simović[2], Aleksandra Tomić[2], Paula López-Revenga[3], Antonia Montilla[3], Francisco Javier Moreno[3] and Dejan Bezbradica[2]

[1]*Innovation Center, Faculty of Technology and Metallurgy, University of Belgrade, Karnegijeva 4, 11000 Belgrade, Serbia;*

[2]*Faculty of Technology and Metallurgy, University of Belgrade, Karnegijeva 4, 11000 Belgrade, Serbia;*

[3]*Grupo de Química y Funcionalidad de Carbohidratos y Derivados, Instituto de Investigación en Ciencias de la Alimentación, CIAL (CSIC-UAM), 28049 Madrid, Spain;*

*Corresponding author: mveljkovic@tmf.bg.ac.rs

Methods

Fourier-Transform Infrared Spectroscopy (FTIR) Analysis. An FTIR analysis of extracted pectic polysaccharides-enriched fractions was performed. FTIR spectra were obtained in a Bruker IFS66v instrument (Bruker, Bremen, Germany). Data were collected in absorbance mode using a frequency range of 4000–400 cm^{-1} and a resolution of 4 cm^{-1} (mid infrared region) with 250 co-added scans.

Determination of Monomeric Composition. Monomeric composition of AIR-RSM and the pectic polysaccharide-enriched fractions was determined using a previously established GC-FID method. In short, samples (20 mg/mL solids) were hydrolyzed with 2 M TFA (final concentration) at 110 °C for 4 h under inert conditions. After hydrolysis, 300 µL aliquots were evaporated, followed by the addition of 400 µL phenyl-*β*-D-glucoside (0.5 mg/mL) as internal

standard, and re-evaporation. Samples were then derivatized and analyzed using an Agilent 7820A GC (Agilent Technologies, USA) equipped with a VF-5HT capillary column (30 m × 0.25 mm × 0.10 µm). The injector and detector temperatures were 280 °C and 385 °C, respectively. Nitrogen was used as carrier gas (1 mL/min), with a temperature program from 120 °C to 280 °C at 3 °C/min. Quantification was based on calibration curves (0.02–2 mg/ml) of reference sugars with internal standard (0.2 mg), using Agilent ChemStation software (Rev. B.04.03).

Based on the monomeric composition, various structural parameters were calculated[1].

The HG content was calculated using the following equation:

$$HG(\%) = GalA - Rha$$

The RG-I backbone was characterized using several parameters, including RGI content, degree of branching (DB-RG-I), and extent of branching (EB-RG-I):

$$RG - I\ content\ (\%) = 2 * Rha + Ara + Gal$$

$$DB\ of\ RG - I = \frac{GalA}{Rha}$$

$$EB\ of\ RG - I = \frac{Ara + Gal}{Rha}$$

The linearity of pectin backbone (LP) was calculated using equation:

$$LP = \frac{GalA}{Rha + Ara + Gal}$$

The purity of obtained pectin (PP) was calculated using equation:

$$PP = \frac{GalA + Rha + Ara + Gal}{Glu + Man}$$

Determination of the Molecular Weight Distribution. Molecular weight (M_w) distribution was determined following a previously reported method by Simović et al [2]. In short, samples (20 mg/mL) were dissolved in distilled water at 50 °C for 30 min, diluted 1:9 with 0.04 M ammonium acetate, filtered, and analyzed by High-Performance size Exclusion

Chromatography with evaporative light scattering detection (HPSEC-ELSD; Agilent Technologies, Boeblingen, Germany). Separation was performed using a TSK-Gel guard column (6.0 mm × 400 mm) and two TSK-Gel columns in series: G5000 PWXL (7.8 mm × 300 mm, 10 μm) and G2500 PWXL (7.8 mm × 300 mm, 6 μm) (Tosoh Bioscience, Stuttgart, Germany). A pullulan standard (M_w 0.342–788 kDa, 0.2–2 mg/mL) was used for calibration.

Results and discussion

Protein content in the samples was determined by the Kjeldahl method.

Table S1. Protein content.

Fraction	**Protein content, %**
AIR-RSM	**42.9 ± 2.5**
DP-RSM	**21.25± 1.7**
Pectic polysaccharides fraction I (PP-I)	**35.08± 1.8**
Pectic polysaccharides fraction II (PP-II)	**23.14± 2.8**
Ca-bound pectic polysaccharides fraction (CaPP-II)	**10.12± 0.9**
Pectic polysaccharides fraction EAE (PP-EAE)	**3.9 ± 0.4**
Ca-bound pectic polysaccharides fraction EAE (CaPP-EAE)	**4 ± 0.5**

FTIR analysis

Fourier-transform infrared (FTIR) spectroscopy, a technique for analysis of functional groups present in polysaccharides, was employed in this study to identify structural differences in the pectic polysaccharide fractions obtained from AIR-RSM using a two-step enzymatic method and additional extraction using ammonium oxalate. The obtained FTIR spectra are presented in Fig. 2. The PP-EAE(24h) and CaPP-EAE(24h) exhibited characteristic absorption bands at 3300, 2930, 1075, and 1030 cm^{-1}, corresponding to inter- and intramolecular O–H stretching, C–H stretching of CH_2 and CH_3 groups, C–OH side groups, and C–O–C vibrations of glycosidic linkages. In general, all pectin samples show absorption bands around 1740 cm^{-1},

1640 cm^{-1} and 1420 cm^{-1} which are attributed to the C=O stretching vibration of methyl esterified carboxyl groups, carboxylate anion (COO^-) stretching vibration, and to the C=O stretching vibration of free carboxyl groups, respectively.[3] The relative portion of 1740 cm^{-1} and 1640 cm^{-1} areas is used to determine the degree of methyl esterification (DM) of the pectin samples. The FTIR spectra of the PP-EAE and CaPP-EAE fractions obtained in this study show only a very weak band at 1740 cm^{-1}, it can be inferred that esterified carboxyl groups are largely absent. This indicates that the extracted pectic polysaccharide fractions possess a low degree of methyl esterification (DM < 10). Ultimately, the bonds within the 800 to 1200 cm^{-1} range (the fingerprint region) are distinctive to each compound and its specific extraction method. The absorption band at 1230 cm^{-1} corresponds to the cyclic C–C stretching within the ring structure of pectin, whereas the characteristic bands at 950 and 780 cm^{-1} are associated with CH_3 rocking vibrations and C–CH and C–OH bending at the C-6 position.[4] Based on FTIR analysis, it can be concluded that the pectic polysaccharides-enriched fractions extracted from AIR-RSM fractions are classified as low-methoxyl (LM) PPs, with a DM below 50%. These LM pectins are capable of forming gels through calcium-mediated cross-linking, rendering them suitable for applications in low-sugar food formulations as well as in biomedical fields such as bioactive compound encapsulation and environmental remediation.[5]

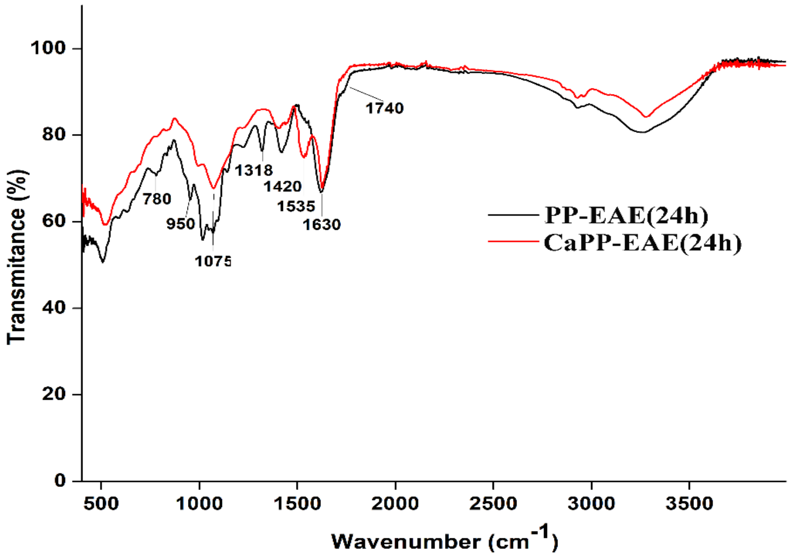

Figure S1. FTIR spectra of the obtained pectin-enriched fractions.

The spectral bands associated with the amide I and II of proteins, typically observed around 1651 cm^{-1} and 1555 cm^{-1}, respectively, were not detected in the PP-EAE sample. However, in the CaPP-EAE fraction, a band at 1535 cm^{-1} was present. This aligns with the protein content in these samples, which is approximately 4%. Nonetheless, the protein content in the pectin samples may be sufficiently high to enhance and promote the interfacial adsorption of pectin at the oil-water interface, thereby bestowing pectic polysaccharides with excellent emulsifying properties.[6]

Prebiotic activity

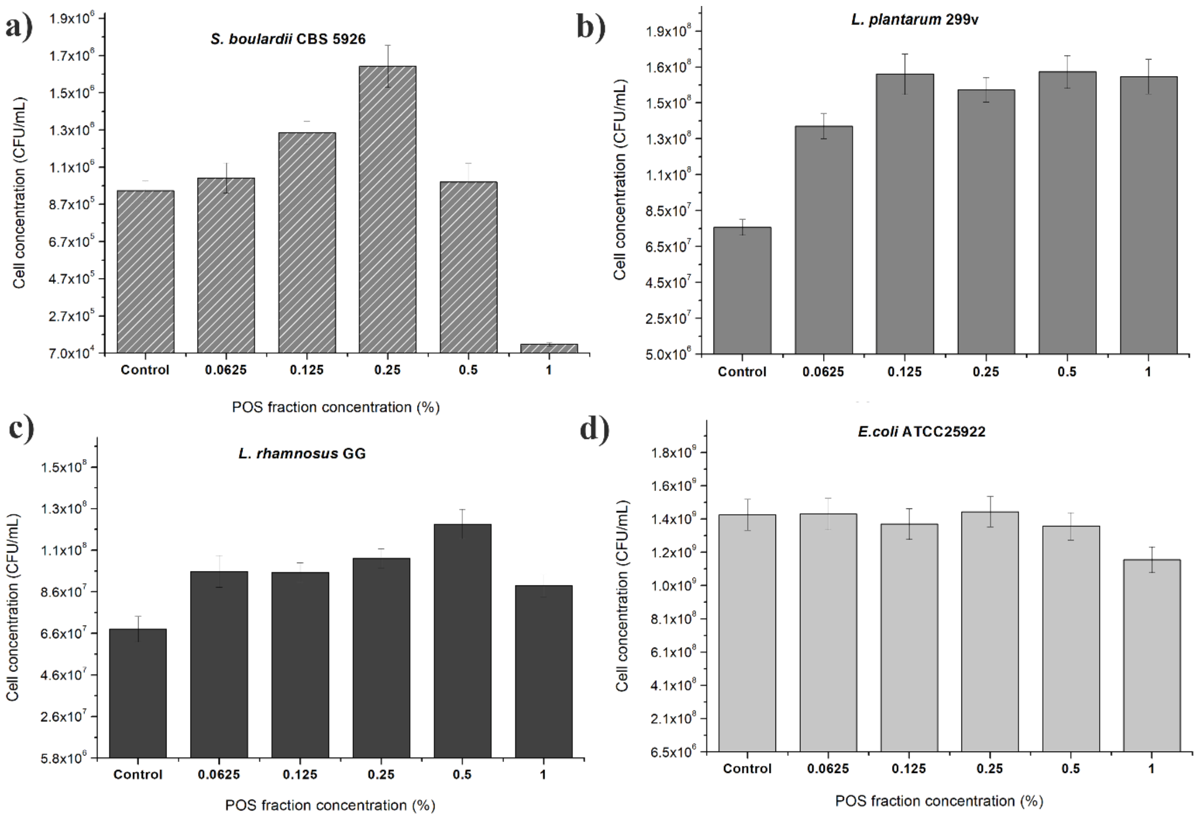

Figure S2. Effect of different POS fraction concentration on the growth of: a) Saccharomyces boulardi CBS 5926, b) Lactiplantibacillus plantarum 299v, c) Lacticaseibacillus rhamnosus GG, and d) Escherichia coli ATCC 25922, after 24 h of incubation.

References

[1] M. Ćorović, A. Petrov Ivanković, A. Milivojević, M. Veljković, M. Simović, P. López-Revenga, A. Montilla, F.J. Moreno, D. Bezbradica, Valorisation of Blackcurrant Pomace by Extraction of Pectin-Rich Fractions: Structural Characterization and Evaluation as Multifunctional Cosmetic Ingredient, Polymers 16(19) (2024) 2779.

[2] M. Simović, K. Banjanac, M. Veljković, V. Nikolić, P. López-Revenga, A. Montilla, F.J. Moreno, D. Bezbradica, Sunflower Meal Valorization through Enzyme-Aided Fractionation and the Production of Emerging Prebiotics, Foods 13(16) (2024) 2506.

[3] F. Jafari, F. Khodaiyan, H. Kiani, S.S. Hosseini, Pectin from carrot pomace: Optimization of extraction and physicochemical properties, Carbohydr. Polym. 157 (2017) 1315-1322.

[4] N. Muñoz-Almagro, A. Ruiz-Torralba, P. Méndez-Albiñana, E. Guerra-Hernández, B. García-Villanova, R. Moreno, M. Villamiel, A. Montilla, Berry fruits as source of pectin: Conventional and non-conventional extraction techniques, Int. J. Biol. Macromol. 186 (2021) 962-974.

[5] I. Ventura, J. Jammal, H. Bianco-Peled, Insights into the nanostructure of low-methoxyl pectin–calcium gels, Carbohydr. Polym. 97(2) (2013) 650-658.

[6] L. Roman, M. Guo, A. Terekhov, M. Grossutti, N.P. Vidal, B.L. Reuhs, M.M. Martinez, Extraction and isolation of pectin rich in homogalacturonan domains from two cultivars of hawthorn berry (Crataegus pinnatifida), Food Hydrocoll. 113 (2021) 106476.
